# Cerebral Venous Thrombosis After a Possible Inadvertent Dural Puncture for Labor Epidural Analgesia

**DOI:** 10.7759/cureus.4822

**Published:** 2019-06-04

**Authors:** Azfar K Niazi, Paul Minko, Kavita K Elliott, Tamer R Ghaly, Sabry Ayad

**Affiliations:** 1 Outcomes Research, Cleveland Clinic, Cleveland, USA; 2 Anesthesiology, Cleveland Clinic Fairview Hospital, Cleveland, USA

**Keywords:** cerebral venous thrombosis, post-dural puncture headache, factor v leiden mutation

## Abstract

Pregnancy is a hypercoagulable state that increases the risk of thrombotic complications. A 32-year-old gravida 4 para 3 (G4P3) had a dural puncture during epidural catheter placement for labor analgesia. A positional headache started after delivery and continued for several days. A week after the delivery, she developed non-positional headaches along with seizures. Magnetic resonance imaging (MRI) and magnetic resonance venography (MRV) lead to the diagnosis of cerebral venous thrombosis (CVT). A factor V Leiden mutation was also found; that was suspected to contribute to the development of CVT along with dural puncture and pregnancy. CVT can present with non-positional headaches a week after the dural puncture.

## Introduction

Pregnancy is a hypercoagulable state that increases the risk of stroke [1]. Ischemic stroke occurs more frequently than hemorrhagic ones. Most of these strokes occur close to the time of delivery, in the third trimester, and at puerperium [2]. Although rare, cerebral venous thrombosis (CVT) has a significant mortality rate of 2%-10% [3-4]. Pregnancy increases susceptibility to thrombosis, and this risk can be amplified by thrombophilias. We present a case of a 32-year-old woman who developed CVT a week after delivery.

## Case presentation

A 32-year-old, gravida 4 para 3, received epidural analgesia for labor and delivery. The patient started complaining of a positional headache after vaginal delivery. The headache was mainly in the frontal region, with some involvement of the back of the head and neck areas, and was associated with severe nausea and vomiting but no diplopia, tinnitus or fever.

**Figure 1:**
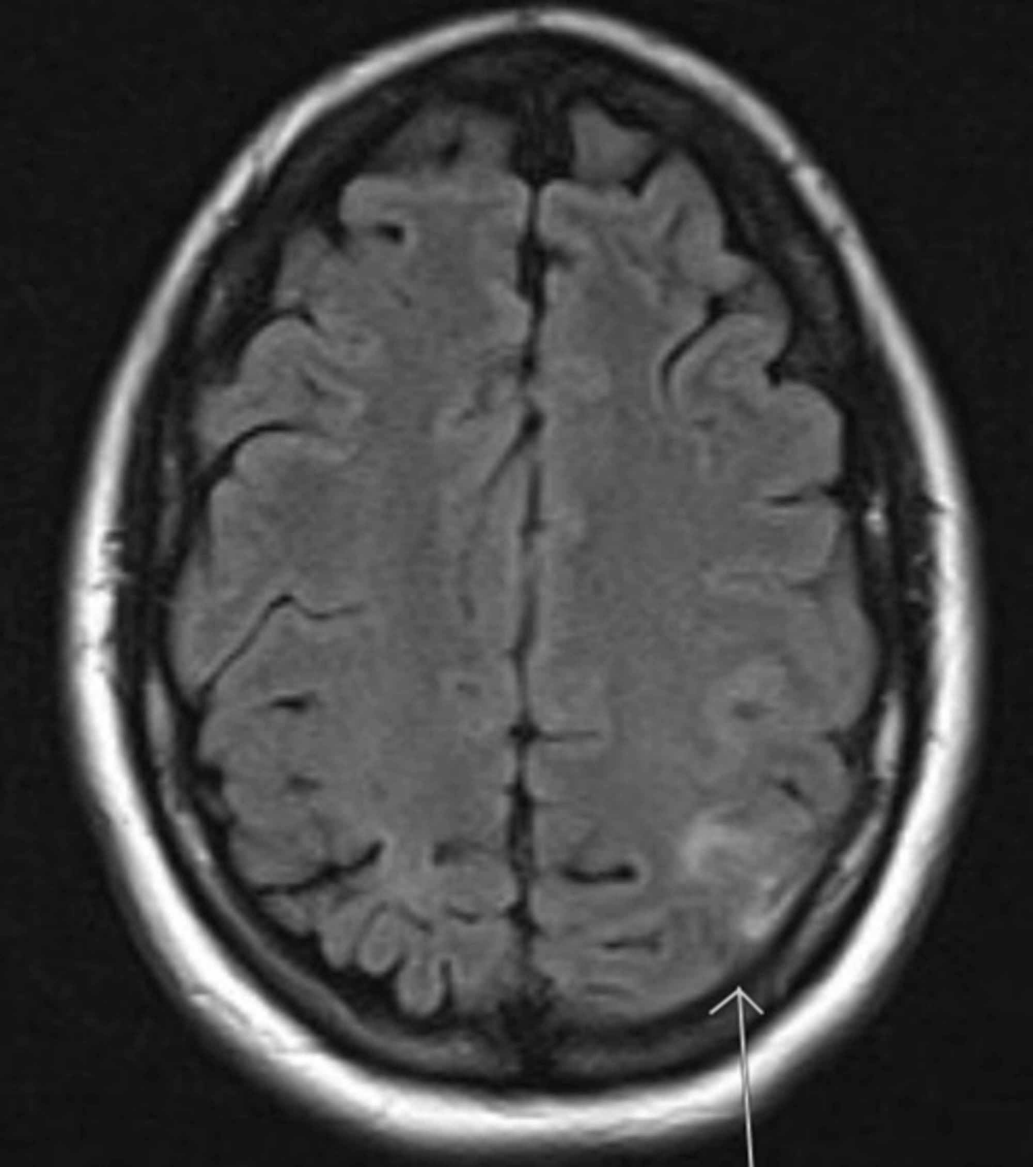
A hyperintense area can be seen on this axial magnetic resonance imaging of the brain (shown with the white arrow)—a prominent sign of cerebral vein thrombosis.

A post-dural puncture headache was diagnosed and the patient refused treatment with an epidural blood patch. The patient did not have any prior history of migraines or hypercoagulable disorders. During the course of hospitalization, before the initiation of epidural analgesia, the patient was adequately hydrated, with a 1000 ml bolus of lactated ringer solution and then a continuous infusion of lactated ringers continued thereafter, at a rate of 125 ml/hour for a total of three liters. The patient was discharged two and a half days after the delivery on acetaminophen and caffeine. The headache was controlled with analgesics, and she did not have any nausea at the time of discharge. Her symptoms improved slightly over the next few days. A week later, the patient started having a non-positional headache and blurred vision followed by tonic-clonic seizures for which she was taken to the emergency department. Eclampsia was ruled out by clinical evaluation, blood pressure monitoring, and laboratory investigations. Computed tomography (CT) revealed no acute pathology. MRI (Figure 1) and MRV of the brain revealed left cerebral vein thrombosis. Coagulation studies showed decreased activated protein C resistance ratio (APC-R). Factor V Leiden genetic testing was performed, which showed patient heterozygous for the R506Q mutation in the factor V gene, also known as Factor V Leiden.

She was immediately started on heparin intravenously, for anticoagulation, and anti-seizure medication, oxcarbazepine. Heparin was stopped and enoxaparin was bridged to warfarin by protocol. She did not experience further seizures but continued to have intermittent headaches. At the six-month follow-up, all of the patient’s symptoms were completely resolved without any sequelae. Warfarin was switched to aspirin. The patient did not show any new symptoms for a year, after which a follow-up MRI was performed that showed cortical volume loss consistent with prior cerebral venous thrombosis. Follow-up electroencephalogram (EEG) was performed and no epileptiform discharges were recorded, therefore, anti-seizure medication was discontinued. After stopping the medication, the patient was followed up for over a year, with no remarkable headaches or seizures. The patient was placed on long-term aspirin prophylaxis.

## Discussion

The association of dural puncture and CVT has been reported in the literature [5-7]. Although the causal relationship could not be established, Guner at al. studied the frequency of cerebral venous thrombosis and dural puncture and found that 19.6% of patients who had a dural puncture in the previous seven days developed CVT [8]. In our case, the development of CVT a week after dural puncture and symptoms, i.e. headache and seizures, are consistent with the published literature [8-9]. The exact mechanism is not known. However, it is thought to occur because of lumbar puncture that reduces the pressure and volume of cerebrospinal fluid, which results in an increase in the volume of cerebral veins and descent of the brain/brainstem [10]. Additionally, Canoe et al. mentioned an approximately 50% reduction in the speed of blood flow in the straight sinus after a lumbar puncture is performed [11].

A post-dural puncture headache is characteristically different from a headache associated with CVT. The mechanism described by the Monro-Kellie-Abercrombie doctrine states that after a dural puncture, the volume and pressure of cerebrospinal fluid are significantly reduced, leading to, first, an increase in the intracranial venous volume and, second, to the descent of the brain and brainstem structures. The venous volume expansion results from venous stasis and dilation of the sinuses and cortical and spinal veins. In addition, with the descent of the brain, the distortion and stretching of the veins occur, leading to aggravation of symptoms in the upright posture [10]. The most common presentation is in the frontal and occipital areas radiating to the neck and shoulders. Other important associated findings include nausea, vomiting, hearing loss, tinnitus, vertigo, dizziness, visual disturbance, and upper and lower limb pain [12]. When CVT develops, the headache does not remain positional [10]. Associated findings may include visual disturbance, papilledema, focal neurological signs, seizures, and coma [13].

More than half of the cases of postpartum headaches are benign [14]. The most common cause of benign headaches is a post-dural puncture headache followed by primary headache syndromes (migraine, cluster, and tension headaches) as the second most common cause [14]. On the other hand, life-threatening headaches are secondary headaches and are caused by complications of anesthesia, intracranial pathology, or pregnancy/delivery [15]. These include intracranial mass, preeclampsia, meningitis, strokes, venous sinus thrombosis, and reversible cerebral vasoconstrictive syndromes [15].

There were other factors that support the diagnosis of CVT in our case. Seizures have been frequently associated with CVT, however, they are not a predictor of long-term morbidity or mortality [16]. The patients most frequently present with symptoms of headache, cerebral hypertension symptoms, seizures, focal neurological signs, and encephalopathy [17]. Factor V Leiden mutation leads to activated protein C resistance that increases the risk of cerebral venous thrombosis [18]. This risk multiplies because pregnancy itself is a hypercoagulable state.

The investigation of choice is MRI/MRV [19]. It should be performed when there is a dural leak and the headache is non-remitting or changing in character. For future pregnancies, the risk of complications does not appear to be correlated with the thrombophilia (Factor V Leiden mutation) itself, but it is correlated to the previous episode of CVT. Prophylaxis with low molecular weight heparin reduces the risk of recurrent thrombosis or bleeding diathesis but their risk of late obstetrical complications remains high [20].

## Conclusions

CVT is a possible complication of dural puncture. It can present with non-positional headache, seizures, and neurological signs. Thrombophilia multiplies the risk further. MRI/MRV should be performed immediately upon suspicion to treat early and avoid complications.
